# Clinical Trends and Hospital Mortality of Transjugular Intrahepatic Portosystemic Shunt (TIPS) in Germany: A Descriptive Analysis Between 2019 and 2023

**DOI:** 10.3390/diagnostics15151902

**Published:** 2025-07-29

**Authors:** Sven H. Loosen, Christian Weigel, Anselm Kunstein, Peter Minko, Gerald Antoch, Johannes G. Bode, Tom Luedde, Christoph Roderburg, Karel Kostev

**Affiliations:** 1 Department of Gastroenterology, Hepatology and Infectious Diseases, University Hospital Düesseldorf, Medical Faculty of Heinrich Heine University Düsseldorf, Moorenstraße 5, 40225 Düsseldorf, Germany; sven.loosen@med.uni-duesseldorf.de (S.H.L.); anselm.kunstein@med.uni-duesseldorf.de (A.K.); johannes.bode@med.uni-duesseldorf.de (J.G.B.); tom.luedde@med.uni-duesseldorf.de (T.L.); christoph.roderburg@med.uni-duesseldorf.de (C.R.); 2 Center for Integrated Oncology Aachen-Bonn-Cologne-Duesseldorf (CIO^ABCD^), Kerpener Straße 62, 50937 Köln, Germany; peter.minko@med.uni-duesseldorf.de (P.M.); antoch@med.uni-duesseldorf.de (G.A.); 3 Department of Diagnostic and Interventional Radiology, University Hospital Düsseldorf, Medical Faculty of Heinrich Heine University Düsseldorf, Moorenstraße 5, 40225 Duesseldorf, Germany; 4 Epidemiology Department, IQVIA GmbH, 60549 Frankfurt, Germany; karel.kostev@iqvia.com

**Keywords:** portal hypertension, TIPS, liver cirrhosis, mortality, German

## Abstract

**Background/Objectives:** The transjugular intrahepatic portosystemic shunt (TIPS) is an established treatment for complications of portal hypertension in patients with liver cirrhosis. While its use has increased and indications have broadened in recent years, recent comprehensive data on patient characteristics, trends, and in-hospital mortality in Germany are lacking. This study aimed to evaluate current clinical patterns and mortality outcomes associated with TIPS. **Methods:** This nationwide cross-sectional study used anonymized hospital data from the German InEK database between 2019 and 2023. TIPS procedures were identified using relevant OPS codes. Patient demographics, liver cirrhosis stage (Child–Pugh), hepatic encephalopathy grade, comorbid conditions, and in-hospital mortality were analyzed descriptively. Analyses were conducted using SAS 9.4. **Results:** A total of 12,905 TIPS procedures were documented. Annual case numbers rose from 2180 in 2019 to 2954 in 2023. Most patients were male (66.3%) and aged 60–74 years. Ascites (68.6%) was the most frequent associated diagnosis, followed by variceal bleeding (16.4%) and hepatorenal syndrome (14.9%). The average hospital stay decreased from 19.6 to 16.8 days. Overall in-hospital mortality was 8.5%, increasing with age (13.0% in ≥75 years), Child–Pugh C cirrhosis (14.9%), PCCL grade 4 (17.6%), hepatorenal syndrome (16.7%), and grade 4 hepatic encephalopathy (56.1%). **Conclusions:** TIPS usage in Germany has increased over the past five years, with a shift toward earlier disease stages. Higher in-hospital mortality in clinically complex patients underscores the importance of careful patient selection and tailored management strategies in high-risk groups.

## 1. Introduction

TIPS placement is an established technique for managing complications of portal hypertension [1]. Portal hypertension can result in potentially life-threatening complications, including acute variceal hemorrhage, refractory ascites, and hepatorenal syndrome [2,3]. With increasing experience, advancements in minimally invasive techniques, and improvements in stent materials, the TIPS procedure has evolved over the years from a salvage therapy to a standard method for effectively reducing portal venous pressure in the management of selected patients with clinically significant portal hypertension [4]. 

With technical progress and increasing evidence, TIPS has improved transplant-free survival and TIPS-associated complications were vastly reduced [5]. Multiple studies have demonstrated that the insertion of a TIPS significantly improves survival rates in patients with recurrent ascites [6]. Furthermore, this procedure has been shown to confer survival benefits in carefully selected patients suffering from refractory ascites and those experiencing variceal bleeding, highlighting its efficacy in managing these severe complications of portal hypertension [7,8].

It is therefore not surprising that a nationwide study in Germany reported an increase in the number of TIPS procedures performed between 2007 and 2018, reflecting the growing use of this intervention in managing complications of portal hypertension [9]. Additionally, it has been shown that reducing portal hypertension through TIPS significantly improves patient outcomes by lowering the risk of complications such as variceal bleeding and refractory ascites, thus enhancing overall prognosis [9].

However, more recent data are lacking for a detailed and nuanced analysis of trends in the past few years. This gap highlights the need for updated studies to provide a clearer understanding of developments in patient outcomes, procedural advancements, and clinical practices related to TIPS in more contemporary settings. The present nationwide study is designed to inquire into the latest developments of TIPS and associated mortality in Germany from 2019 to 2023.

## 2. Materials and Methods

### 2.1. Data Source

This cross-sectional study was based on data from the Institute for Hospital Remuneration (InEK, Institut für das Entgeltsystem im Krankenhaus) data browser. This browser covers all hospitals in Germany and contains comprehensive information transmitted by German hospitals to InEK in a standardized format, in accordance with Section 21 of the German Hospital Compensation Act (KHEntgG). To ensure that no conclusions could be drawn about individual patients through searches in the InEK data browser, the case data were limited to what was necessary for the analysis (including a few demographic details along with diagnoses and procedures) and were also modified. For instance, instead of specific ages, only age categories were available. Evaluations of categories or subgroups containing fewer than four data entries were not displayed to prevent the identification of individuals for data protection reasons.

### 2.2. Study Population

This study includes patients with a documentation of TIPS insertion between January 2019 and December 2023. TIPS was selected according to the German procedure coding system (OPS) codes 8-839.81, 8-839.82, 8-839.83, 8-839.85, 8-839.87, 8-839.88, 8-839.89, 8-839.8a, 8-839.8x, and 8-839.8. Based on the individual secondary diagnoses, TIPS patients were classified in those with and without liver cirrhosis (ICD-10: K70.3, K74).

### 2.3. Study Outcome

The descriptive study aimed to estimate the yearly frequency of TIPS in the time between January 2019 and December 2023 and to describe the characteristics of TIPS patients including age groups, sex (female, male), patient clinical complexity level (PCCL), mortality rate, proportion of patients with hepatic encephalopathy (ICD-10: K72.7) diagnosis, liver cirrhosis Child–Pugh stages, as well as secondary diagnoses associated with TIPS indications including ascites (ICD-10: R18), hepatorenal syndrome (ICD-10: K76.7), and variceal bleeding (ICD-10 I98.3). Furthermore, hepatic encephalopathy were classified as grade 1 (ICD-10: K72.71), grade 2 (ICD-10: K72.72), grade 3 (ICD-10: K72.73), grade 4 (ICD-10: K72.74), and unspecified (ICD-10: K72.79).

Finally, the hospital mortality rate was calculated for age groups, women and men, PCCL categories, hepatic encephalopathy stages, and liver cirrhosis Child–Pugh stages. Mortality rate was calculated as the number of patients with death records divided by the total of patients in each category (i.e., age groups, women and men, PCCL categories, hepatic encephalopathy stages, and liver cirrhosis Child–Pugh stages).

All analyses were performed using SAS 9.4 (SAS institute, Cary, NC, USA).

## 3. Results

### 3.1. Patient Characteristics and Clinical Trends of TIPS Placement in Germany

**Figure 1:**
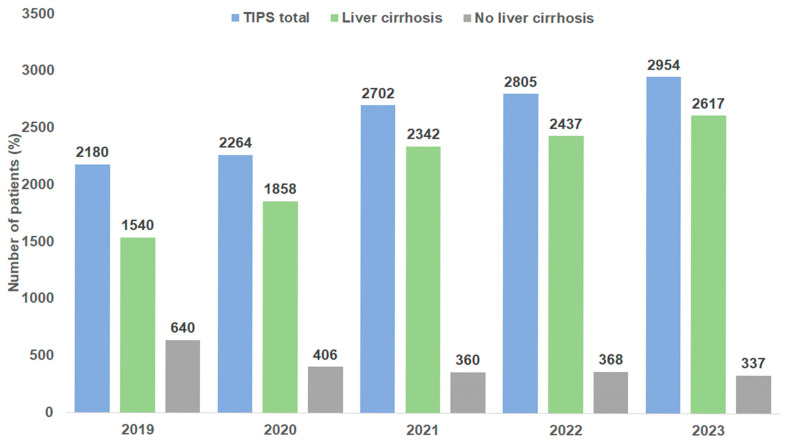
Number of patients with TIPS placement in Germany by year.

**Figure 2:**
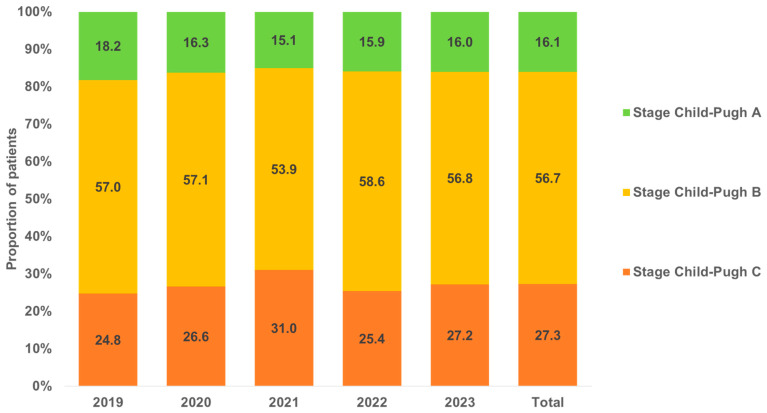
Child–Pugh stages of liver cirrhosis in TIPS patients by year.

**Figure 3:**
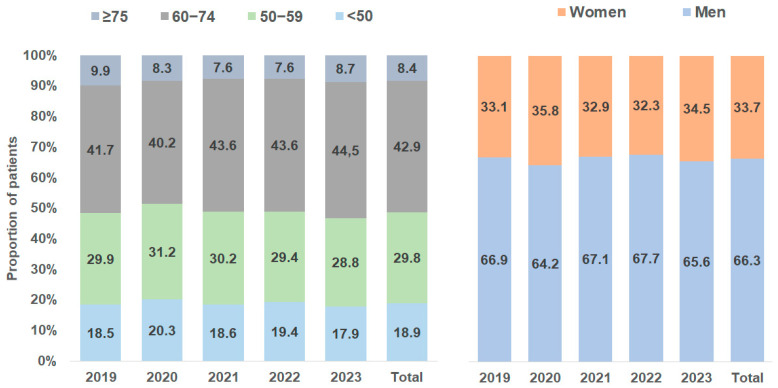
Age and gender of TIPS patients by year.

A total of 12,905 TIPS placements were documented in Germany between 2019 and 2023. We observed a strong increase in annual cases from 2180 in 2019 to 2954 in 2023 (Figure 1). The majority of patients (83.6%) had an underlying diagnosis of liver cirrhosis. This proportion increased during the observation period from 70.6% in 2019 to 88.6% in 2023 (Figure 1). In patients without cirrhosis, the three most common diagnoses were liver cell carcinoma (25.2%), Budd–Chiari syndrome (17.8%), and primary biliary cholangitis (9.2%). Figure 2 shows the Child–Pugh stages of liver cirrhosis for TIPS patients by year. Most patients (56.7%) had a Child–Pugh stage B, followed by 27.3% of patients with a Child–Pugh C stage; 16.1% of patients had a Child–Pugh stage A (Figure 2). The proportions of stages did not linearly change during the study period. In total, 66.3% of patients were male. The proportion of male patients ranged between 64.2% (2020) and 67.7% (2022). In terms of patient’s age, 18.9% of patients were <50 years, 29.8% were between 50 and 59 years, 42.9% were 60–74 years, and 8.4% were 75 years and older. No changes in age and sex distribution were observed between 2019 and 2023 (Figure 3).

### 3.2. Secondary Diagnoses Associated with TIPS Indications and Patient Clinical Complexity Level (PCCL)

**Figure 4:**
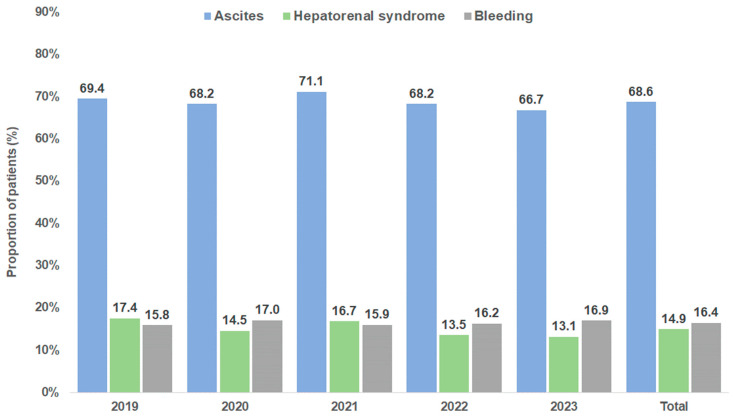
Secondary diagnoses associated with TIPS indications.

Although the database does not contain precise information on the indication for TIPS placement, the pre-defined secondary diagnoses (ascites, variceal bleeding, and hepatorenal syndrome) that are known indications for TIPS were analyzed. The most frequently coded diagnosis was ascites (68.6%), followed by variceal bleeding (16.4%), and hepatorenal syndrome (14.9%). The proportions of the three pre-defined diagnoses over time are shown in Figure 4.

**Figure 5:**
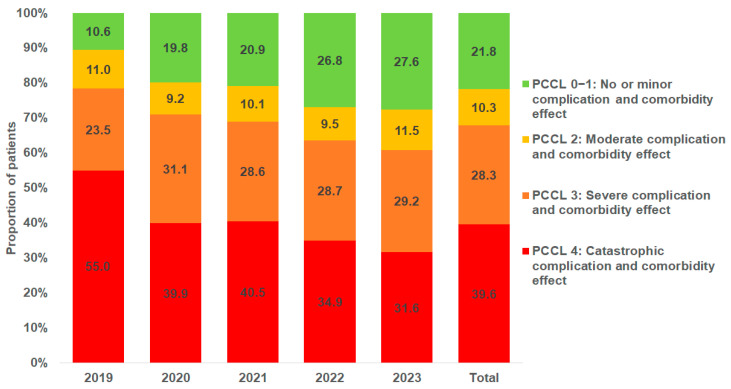
Patient clinical complexity level (PCCL) of patients with TIPS placement in Germany by year.

The patient clinical complexity level (PCCL) is a metric used in healthcare settings and in hospital reimbursement systems, to individually assess the complexity of a patient’s clinical condition. It reflects the overall severity of a patient’s illness based on their comorbidities and complications. In our cohort, the majority of TIPS patients were either classified as “catastrophic complication and comorbidity effect” (PCCL 4, 39.6%) or “severe complication and comorbidity effect” (PCCL 3, 28.3%). Only 21.8% of patients had a moderate and 10.3% no or minor complication and comorbidity effect (PCCL 2 and 1). Figure 5 shows the PCCL distribution by year. The proportion of patients with a PCCL of 4 decreased over time from 55.0% in 2019 to 31.7% in 2023, while the proportion of patients with no or only minor complications and comorbidities (PCCL 1 or 2) increased from 10.6% in 2019 to 27.7% in 2023.

### 3.3. Clinical Course and Hospital Mortality After TIPS Placement

The mean length of hospitalization following TIPS placement was 19.6 days in 2019, 18.2 days in 2020, 17.8 days in 2021, 17.3 days in 2022, and 16.8 days in 2023. Of all 12,905 TIPS patients, 1093 (8.5%) died during the hospital stay. The annual hospital mortality rate varied between 8.0% and 9.2% without a linear trend over time. We next evaluated the hospital mortality in different clinical subgroups in order to identify potential factors that are associated with an increased hospital mortality. Table 1 shows the overall mortality rates over the whole study period (2019–2023). Mortality rates showed a stepwise increase with patients’ age from only 6.5% in patients aged <50 years up to 13.0% in patients aged ≥75 years (Table 1). Mortality rates were comparable between male (8.2%) and female (9.0%) patients. Patients with Child–Pugh A and B liver cirrhosis had a hospital mortality of 3.6% and 5.5% only, while hospital mortality was 14.9% among patients with Child–Pugh C liver cirrhosis (Table 1). TIPS patient with ascites had a hospital mortality of 8.2% that was numerically lower compared to patients with HRS (16.7%) or variceal bleeding (14.6%, Table 1). Moreover, the patient clinical complexity level (PCCL) was associated with hospital mortality. In patients with a PCCL of 1, hospital mortality was only 0.7% but showed a stepwise increase up to 17.6% among patients with a PCCL of 4 (Table 1). Finally, we observed an increasing hospital mortality among patients with hepatic encephalopathy (HE) that ranged from 8.4% in TIPS patients with grade 1 HE to 56.1% in patients with grade 4 HE (Table 1).

## 4. Discussion

The present nationwide study using the InEK registry clearly demonstrates that the insertion of TIPS stents was increasingly performed in the five observed years in Germany, which is in line with another report on German patients pointing out that after the publication of the Baveno V consensus report and the subsequent recommendation in German guidelines for preemptive TIPS for gastroesophageal variceal bleeding and TIPS implementation as first-line treatment in patients with refractory or recurrent ascites [10], TIPS utilization has strongly increased [9]. Of note, this trend was observed only in patients with liver cirrhosis while, in the same period of time, in patients without liver cirrhosis the number of TIPS has dropped from 640 to 337 per year. Interestingly, the proportion of the different indications remained stable over time, with uncontrolled ascites being the most important reason for TIPS insertion. Due to the lack of individual follow-up data, no analysis on TIPS revisions was possible; however, in a similar cohort of patients, TIPS revision contributed around 10% of all TIPS treatments [9].

In our analysis, a TIPS associated mortality of 8.5% was observed, which is in line with previous reports [9,11]. TIPS insertion was associated with an extended hospital stay, with some patients remaining hospitalized for up to 16 days, which is notably long. This prolonged length of stay may be attributed to several factors, including the complexity of the underlying liver disease, the management of post-TIPS complications such as hepatic encephalopathy or acute kidney injury, and the need for close monitoring after the procedure. The extended hospitalization not only impacts patient outcomes but also places a significant burden on healthcare resources. With regard to specific subgroups, the patients’ sex did not affect mortality. In sharp contrast, our findings once again highlight the significant impact of age on post-procedural mortality. Specifically, our analysis showed that while patients under the age of 50 had a relatively low mortality rate following TIPS insertion, mortality rates rise dramatically in those patients over 75 years of age. Interestingly, our data on a mortality of 13.0% in patients older than 75 years very well reproduce recent data from Gu et al. reporting a mortality of 16.14% in patients > 80 years [11]. This age-related increase in mortality is concerning and raises important questions about the appropriateness of TIPS in this subgroup. Since factors such as comorbidities, frailty, and reduced physiological reserve in elderly patients likely contribute to this elevated risk, these elements should be thoroughly assessed before recommending TIPS for older individuals [9]. In line, a recent prospective analysis suggested that TIPS should not be systematically precluded in patients older than 70 years but additional factors including serum creatinine and sodium should rather be used for clinical decision-making [12]. Similarly to age, in our analysis the individual Child–Pugh score turned out to be an important prognostic marker in patients receiving TIPS. Due to the limitations of the InEK database, we are unable to further analyze which specific value of the Child–Pugh score is driving the prognostic power of the score in the context of TIPS. However, by using a different database featuring German patients, Gu et al. identified hypoalbuminemia as well as hepatic encephalopathy as independent prognostic markers after TIPS insertion [9]. Overall, this calls for a nuanced approach for patient selection, where the potential benefits of TIPS must be carefully weighed against the heightened risks in elderly patients.

Interestingly, 8.4% of patients undergoing TIPS were diagnosed with lower-grade hepatic encephalopathy (HE), specifically grade 1. Our data, in line with previous studies [13,14,15,16,17], show that patients with HE grade 1 had slightly lower mortality compared to the overall mortality of TIPS patients, suggesting that lower-grade HE does not negatively impact outcomes. In fact, these patients demonstrated significantly better in-hospital survival after TIPS insertion compared to those who did not undergo the procedure. This supports the notion that pre-TIPS HE, particularly in HE grade 1, is not a reliable predictor of survival and should not be viewed as a contraindication to TIPS. However, our data also confirm that high-grade HE, particularly grades 3 and 4, is a strong predictor of mortality, reinforcing that severe or uncontrolled HE should be considered a relative contraindication, especially when no clear precipitating factors are identified [1]. For patients with refractory ascites, the survival benefits of TIPS often outweigh the risk of developing post-TIPS HE, which may be mitigated by the prophylactic use of rifaximin and the implementation of smaller-diameter covered TIPS stents to reduce the incidence of post-TIPS HE [18,19]. 

As previously noted, both our data and findings from other studies demonstrate a clear increase in the overall number of TIPS procedures performed in recent years. Interestingly, we observed a decrease in the proportion of patients with a PCCL of 4 over time, from 55.0% in 2019 to 31.7% in 2023, while the proportion of patients with no or only minor complications and comorbidities (PCCL 1 or 2) increased from 10.6% to 27.7% during the same period. This trend is likely attributable to an expansion of the indications for TIPS, allowing patients to undergo the procedure earlier in the course of their disease. This may represent an important development, as significantly higher mortality rates are clearly associated with more complex cases—such as those involving advanced liver disease or multiple comorbidities—corresponding to higher PCCL stages. Moreover, it may reflect an improved understanding in recent years of which patients are unlikely to benefit due to an unfavorable risk–benefit profile. Although this trend could indicate a positive shift in clinical practice, the criteria for patient selection must be rigorously re-evaluated to ensure that TIPS is reserved for those who are most likely to derive benefit from the procedure.

Thus, further studies are needed to refine the risk stratification models for TIPS candidates, particularly in patients with severe hepatic dysfunction, multiple comorbidities, or advanced stages of liver disease. A more cautious approach in expanding the use of TIPS may help mitigate the increased mortality rates in this vulnerable patient group and optimize outcomes by ensuring that only appropriately selected patients receive the procedure. For precise risk stratification in the context of advanced liver disease, particularly cirrhosis, there are various prognostic scores designed to estimate patient mortality which can be used to make a proper selection among patients in context of TIPS implantation. The Child–Pugh score has long been the standard for assessing the prognosis of patients with cirrhosis. Additionally, the MELD score (including MELD-Na and MELD 3.0) has been widely used not only for predicting short-term mortality in patients on the liver transplant waiting list, but also increasingly in patients with decompensated cirrhosis outside of the transplant setting. The Chronic Liver Failure Consortium scores, CLIF-C ACLF and CLIF-C AD, were developed as tailored tools to predict the prognosis of patients with acute-on-chronic liver failure or acute decompensation of cirrhosis [20,21]. Recently, the Freiburg Index of Post-TIPS Survival (FIPS) was developed, offering a significantly improved prognostic assessment for patients with cirrhosis undergoing TIPS implantation due to refractory ascites, secondary prophylaxis of variceal bleeding, or acute decompensation [22,23].

Our study has some limitations. First, the data were only available as aggregated tables and not on an individual patient level, with a consequence that only descriptive but no statistical analyses (i.e., regression analyses) were possible. Second, the key information in the present study derived from the InEK database consists of OPS codes, which are used within the framework of the German DRG system. Due to the complexity of the German DRG system, inaccuracies in the primary coding cannot be ruled out. However, since the hospital reimbursement system in Germany is based on the DRG system, the accuracy of the data is assumed. Third, the database does not include lab data (i.e., to calculate the MELD (Model for End-Stage Liver Disease) score), socioeconomic, or lifestyle variables (alcohol and smoking behavior). Fourth, given the present data, it should be noted that due to the focus on the investigated parameters per patient in the InEK database, no follow-up data or further clinical information for individual patients and subgroups are available. This focus was necessary to ensure that no conclusions could be drawn about individual patients within the study. However, this limitation means that no statements can be made regarding relevant predictors for increased risk; for example, the presence of important clinical conditions, such as hepatic encephalopathy, before or after the TIPS procedure cannot be differentiated based on the available data. Furthermore, it must be acknowledged that no information regarding the cause of in-hospital mortality can be derived from the data used, highlighting the necessity for further analysis of more complex datasets to enable concrete conclusions about the management and course of patients after the TIPS procedure.

In conclusion, this comprehensive analysis of nationwide epidemiological data from the InEK registry on TIPS placement in Germany over a 5-year span demonstrates promising developments, with a marked increase in the number of patients benefiting from TIPS insertion. The data suggest that TIPS procedures are generally safe, in particular when performed in low-risk patients. Despite these positive findings, there remains a need for larger, well-designed prospective studies to further refine our understanding of the futility criteria associated with TIPS insertion, ensuring that patient selection is optimized for better outcomes.

## Figures and Tables

**Table 1:** Mortality rate of TIPS patients.

Patient Group	Deceased Patients (N)	Mortality Rate (%)
TIPS total	1093	8.5%
Liver cirrhosis	757	7.0%
No liver cirrhosis	336	15.9%
**Age**		
<50 years	159	6.5%
50–59 years	291	7.6%
60–74 years	503	9.1%
≥75 years	140	13.0%
**Sex**		
male	701	8.2%
female	392	9.0%
**PCCL**		
1	19	0.7%
2	25	1.9%
3	151	4.1%
4	898	17.6%
**Indications**		
Ascites	725	8.2%
Hepatorenal syndrome	322	16.7%
Bleeding	309	14.6%
**Stage of liver cirrhosis**		
Child–Pugh A	56	3.6%
Child–Pugh B	305	5.5%
Child–Pugh C	396	14.9%
**Grades of hepatic encephalopathy**		
Hepatic encephalopathy total	515	18.6%
Hepatic encephalopathy Grade 1	79	8.4%
Hepatic encephalopathy Grade 2	114	15.2%
Hepatic encephalopathy Grade 3	139	26.8%
Hepatic encephalopathy Grade 4	106	56.1%
Hepatic encephalopathy Grade Unknown	77	20.8%

## Data Availability

The data that support the findings of this study are available on request from the corresponding authors.
